# Mutational Studies of Putative Biosynthetic Genes for the Cyanobacterial Sunscreen Scytonemin in *Nostoc punctiforme* ATCC 29133

**DOI:** 10.3389/fmicb.2016.00735

**Published:** 2016-05-18

**Authors:** Daniela Ferreira, Ferran Garcia-Pichel

**Affiliations:** School of Life Sciences, Arizona State University, TempeAZ, USA

**Keywords:** scytonemin, *scy* genes, cyanobacteria, *Nostoc punctiforme*, cyanobacterial deletion mutants

## Abstract

The heterocyclic indole-alkaloid scytonemin is a sunscreen found exclusively among cyanobacteria. An 18-gene cluster is responsible for scytonemin production in *Nostoc punctiforme* ATCC 29133. The upstream genes *scyABCDEF* in the cluster are proposed to be responsible for scytonemin biosynthesis from aromatic amino acid substrates. *In vitro* studies of ScyA, ScyB, and ScyC proved that these enzymes indeed catalyze initial pathway reactions. Here we characterize the role of ScyD, ScyE, and ScyF, which were logically predicted to be responsible for late biosynthetic steps, in the biological context of *N. punctiforme*. In-frame deletion mutants of each were constructed (Δ*scyD*, Δ*scyE*, and Δ*scyF*) and their phenotypes studied. Expectedly, Δ*scyE* presents a scytoneminless phenotype, but no accumulation of the predicted intermediaries. Surprisingly, Δ*scyD* retains scytonemin production, implying that it is not required for biosynthesis. Indeed, *scyD* presents an interesting evolutionary paradox: it likely originated in a duplication event from *scyE*, and unlike other genes in the operon, it has not been subjected to purifying selection. This would suggest that it is a pseudogene, and yet *scyD* is highly conserved in the scytonemin operon of cyanobacteria. Δ*scyF* also retains scytonemin production, albeit exhibiting a reduction of the production yield compared with the wild-type. This indicates that ScyF is not essential but may play an adjuvant role for scytonemin synthesis. Altogether, our findings suggest that these downstream genes are not responsible, as expected, for the late steps of scytonemin synthesis and we must look for those functions elsewhere. These findings are particularly important for biotechnological production of this sunscreen through heterologous expression of its genes in more tractable organisms.

## Introduction

**FIGURE 1:**
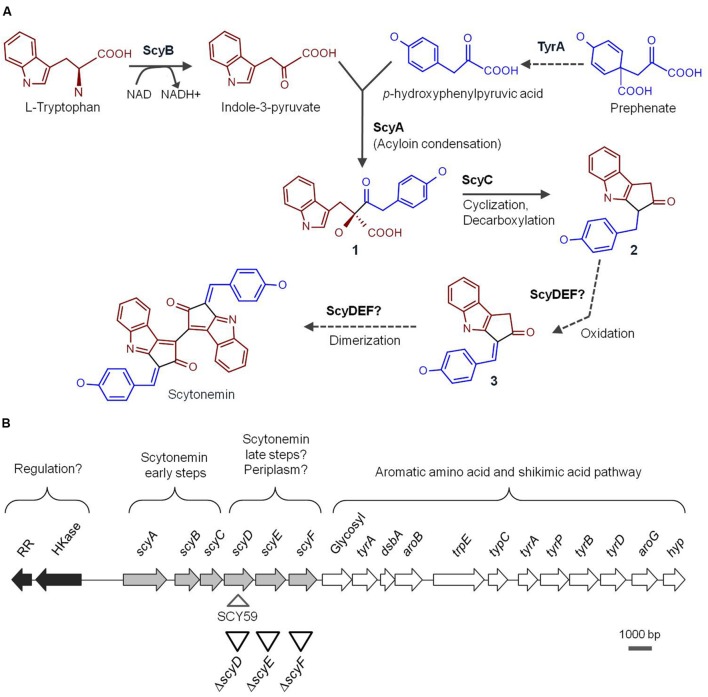
**(A)** Proposed scytonemin biosynthetic pathway. The names of the enzymes (proved and speculative) involved in the process are written next to the arrows that symbolize reactions catalyzed by them. Bold numbers label the intermediate compounds. **(B)** Physical map of the *Nostoc punctiforme* genomic region containing the 18-gene cluster associated with scytonemin production. The arrows represent ORFs and are filled according to the following functional categories: white are scytonemin core genes, light gray are genes associated with aromatic amino acid biosynthesis, and dark gray are putative regulators. Upside down triangles depict approximate locations of the in-frame deletion mutants constructed in this study while the right side up triangle shows the scytoneminless insertion mutant, SCY 59, obtained previously (Soule et al., 2007) and used for comparison purposes in this study.

In order to succeed in habitats exposed to high exposure to UV radiation, such as soil and rock surfaces, cyanobacteria must use various UV radiation defense mechanisms. Sunscreens serve as passive preventative mechanisms that allow them to intercept UV before it reaches targets in the cellular machinery (Gao and Garcia-Pichel, 2011). Scytonemin, found exclusively among cyanobacteria, is a brownish-yellow, lipid-soluble pigment that is excreted and accumulates in the extracellular matrix of cells exposed to UV-A radiation (315-400 nm) (Garcia-Pichel and Castenholz, 1991; Garcia-Pichel et al., 1992; Rastogi et al., 2013; Rastogi and Incharoensakdi, 2014). Structurally unique among natural products, scytonemin is a homodimeric indole-alkaloid, with a molecular mass of 544 g mol^–1^, composed of two heterocyclic units (cyclopentyl[b]indole) that are symmetrically connected through a carbon–carbon bond (Proteau et al., 1993) (**Figure 1A**). The complex ring structures allow a strong absorption in the UV-A-violet-blue range (325–425 nm), with a maximum of 384 nm in acetone and around 370 nm *in vivo* (Garcia-Pichel and Castenholz, 1991; Proteau et al., 1993). Scytonemin has also received attention for its anti-inflammatory and anti-proliferative activities, acting as an inhibitor of human *polo*-like kinase 1 (Stevenson et al., 2002; Zhang et al., 2013). The increasing interest of the scytonemin use for pharmaceutical applications (Soule and Garcia-Pichel, 2014; Rastogi et al., 2015), might lead to a higher demand of this pigment. Thus, a better understanding of its biological synthetic pathway will be essential for future heterologous expression with an eye on biotechnological production, e.g., in *Escherichia coli* (Malla and Sommer, 2014).

Proteau et al. (1993), with the discovery of the scytonemin eight ring structure, hypothesized that this molecule was formed by the condensation of tryptophan- and phenylpropanoid-derived subunits. The construction of a *Nostoc punctiforme* ATCC 29133 (PCC 73102) scytoneminless mutant strain, Soule et al. (2007), opened the door to the study of the molecular genetics of scytonemin biosynthesis. In order to locate the gene(s) involved in scytonemin biosynthesis, a *N. punctiforme* mutant unable to produce scytonemin was generated by random transposon insertion. The mutation specific site was traced to the open reading frame (ORF) Npun_R1273 (later assigned as *scyD*), which was part of a gene cluster involving 18 contiguous ORFs (Npun_R1276 to Npun_R1259). A closer analysis of the cluster revealed that six of these genes, *scyABCDEF* (Npun_R1276 to Npun_R1271), coded for novel proteins with unknown function, while the rest of genes located downstream coded for redundant orthologs of enzymes in the aromatic amino acid pathway, tryptophan and *p*-hydroxyphenyl-pyruvate (Soule et al., 2007, 2009b). Therefore, it was suggested that the Scy proteins were probably involved in the assembly of scytonemin from central metabolites, while the downstream region should be responsible for the delivery of biosynthetic precursors (**Figure 1B**). Comparative genomics studies with other strains have shown that the 18-gene scytonemin cluster of *N. punctiforme* is reasonably well conserved among cyanobacteria (Sorrels et al., 2009; Soule et al., 2009b). Furthermore, transcriptional studies demonstrated that, in *N. punctiforme*, these genes are up-regulated under exposure to UV-A radiation (Sorrels et al., 2009; Soule et al., 2009a), and co-transcribed as a single operon (Soule et al., 2009a).

The involvement of ScyA, ScyB, and ScyC in the early stages of the scytonemin assembly was effectively demonstrated *in vitro* (Balskus and Walsh, 2008, 2009). ScyB (Npun_R1275), a leucine dehydrogenase homolog, catalyzes one of the first biosynthetic steps by oxidative deamination of L-tryptophan to provide indole-3 pyruvic acid (**Figure 1A**). TyrA (Npun_R1269), a putative prephenate dehydrogenase, is thought to be responsible for the oxidation of prephenate to *p*-hydroxyphenylpyruvic acid (Gao and Garcia-Pichel, 2011). Subsequently ScyA (Npun_R1276), a thiamin-dependent enzyme, mediates the acyloin coupling of indole-3 pyruvic acid and *p*-hydroxyphenylpyruvic acid, producing the labile β-ketoacid compound 1 (**Figure 1A**) (Balskus and Walsh, 2008). ScyC (Npun_R1274) catalyzes the cyclization and decarboxylation of compound 1 to form a ketone (compound 2, **Figure 1A**). The compound yielded by ScyC is then just one oxidation state away from compound 3, a potential scytonemin monomeric precursor (Balskus and Walsh, 2009) (**Figure 1A**). Recently, heterologous expression of the *N. punctiforme scy* genes in *E. coli* corroborated these findings (Malla and Sommer, 2014). Upon supplementation of tryptophan and tyrosine, an *E. coli* BL21 strain carrying both *scyA* and *scyB* genes was able to produce a decarboxylated version of the ScyA product reported by Balskus and Walsh (2008). When *scyC* was expressed together with *scyA* and *scyB*, an accumulation of compound 3 (together with other shunt products) was observed. Interestingly, however, the same compounds were produced when expressing all six *scy* genes in *E. coli*.

A previous study in our group predicted that the enzymes encoded by *scyD*, *scyE*, and *scyF* could be periplasmic proteins, and would logically be involved in the late steps of the scytonemin biosynthetic pathway (**Figure 1B**) (Soule et al., 2009b). Consequently, it was speculated that scytonemin biosynthesis is compartmentalized in the cell, with the monomer synthesis and initial condensation occurring in the cytoplasm, and the later reactions taking place in the periplasm, before the pigment is excreted (Soule et al., 2009b; Gao and Garcia-Pichel, 2011). In this study, we evaluate and discuss the role of ScyD, ScyE, and ScyF in the scytonemin biosynthetic pathway of *N. punctiforme*.

## Materials and Methods

### Cultures and Culture Conditions

All experiments in this paper were conducted with a wild-type (WT) *N. punctiforme* strain ATCC 29133 (PCC 73102) derivate, UCD 153, that displays a dispersed growth under shaking culture conditions and a higher frequency of gene replacement by conjugal transfer compared to the original isolate, and mutants derived from it. WT and mutant strains of *N*. *punctiforme* were grown under standard culture conditions as previously described Campbell et al. (2007) in liquid Allen and Arnon medium (Allen and Arnon, 1955), diluted fourfold (AA/4), and on solidified AA medium plates. When necessary, the medium was supplemented with 2.5 mM NH_4_Cl buffered with 5 mM MOPS (pH 7.8). Neomycin at 25 μg ml^–1^ was used for the selection and maintenance of transformed *N*. *punctiforme* strains. *Escherichia coli* strains and derivatives were grown in liquid or solid Lysogeny Broth (LB) supplemented with antibiotics at the following concentrations: kanamycin 25 μg ml^–1^, chloramphenicol 30 μg ml^–1^, and ampicillin 100 μg ml^–1^.

### Construction of Mutants

All chromosomal mutations were in-frame deletions generated by PCR using *N. punctiforme* genomic DNA. Primers were designed to amplify DNA to the right and left of the deletion (≥2.0 kb on each side to allow for homologous recombination) with the primers adjacent to the deletion containing overlapping sequences (see Supplementary Table S1 for oligonucleotide details and gene deletions). PCR products from above were mixed and allowed to anneal via overlapping sequences, and a subsequent PCR produced a product with the deletion. These mutations should only affect the deleted portion, and not cause polar effects on downstream genes. The products were sequenced to ensure fidelity. The *scyE* and *scyF* deletions were first cloned into pUC18 and then digested with XbaI and Eco53kI to be subcloned into the *sacB*-containing pRL278 (Cai and Wolk, 1990) digested with the same enzymes, while the *scyD* deletion was directly cloned into pRL278 as a XhoI fragment using restriction sites introduced on the primers. The gene deletion suicide-plasmids were introduced into WT *N*. *punctiforme* via triparental conjugation using *E. coli* strains as carriers of recombinant plasmids as described elsewhere (Cohen et al., 1994), with two exceptions: (i) no sonication was performed to fragment *N. punctiforme* filaments prior to conjugation, and (ii) conjugative plates were not supplemented with CO_2_-enriched air. Single recombinant strains, in which the suicide-plasmid was integrated into the genome, were selected and maintained in medium containing neomycin. Strains in which the suicide-plasmid and WT gene were eliminated via a second recombination event were selected on AA plates containing 5% (w/v) sucrose. PCRs confirmed elimination of the WT gene and replacement with the respective inactivated gene (Supplementary Figure S1).

### Characterization of Δ*scyD*, *ΔscyE*, and Δ*scyF* Mutant Strains

*Nostoc punctiforme* biomass from WT and derived deletion mutants was tested for the ability to produce scytonemin. Cultures grown for 3–4 days, with a chlorophyll *a* content of approximately 1–2 μg ml^–1^, were concentrated to 50 μg of chlorophyll *a* ml^–1^ and spread over polycarbonate membrane filters (0.4 μm pore size), which were placed floating on liquid medium filled glass petri dishes, as previously described (Garcia-Pichel and Castenholz, 1991). The cells were exposed to white light (7 W m^–2^) supplemented with UV-A, provided by 20 W black-light fluorescent tubes (General Electric) at an intensity of 10 W m^–2^ as described in (Garcia-Pichel et al., 1992), for a minimum of five continuous days. To ensure a correct evaluation of a scytoneminless phenotype, longer exposure times of 8 days were applied as well, and at least three experimental replicates were performed. Following UV-A exposure, the cells were harvested and the lipid-soluble pigments were extracted from whole cells in the same volume of 100% acetone. Extracts were analyzed with a spectrophotometer for absorption between 330 and 730 nm, a strong absorption peak at 384 nm indicating that scytonemin had accumulated in the cells (Garcia-Pichel and Castenholz, 1991). Additionally, cultures were observed microscopically for changes in extracellular pigmentation: the presence/absence of scytonemin was easily detected by color around the cells and confirmed the spectrophotometry and HPLC data; possible colored pigment precursors were not visible (Supplementary Figure S2).

For the comparative quantification of scytonemin accumulation by the WT, *ΔscyD*, and *ΔscyF* cells, 50 μl of concentrated acetone extracts from cells exposed to UV-A for 6 days were analyzed by high pressure liquid chromatography (HPLC) using the same procedure as Karsten and Garcia-Pichel (1996). Carotenoids, chlorophyll *a*, and scytonemin were monitored in the chromatograms at 384 nm, but spectra were also recorded continuously between 190 and 700 nm using an Agilent 1100 HPLC system with an online photodiode array detector. The individual pigments were identified by their characteristic absorption maxima corresponding to the appropriate retention time (chlorophyll *a* at 17.5 min and scytonemin at 2.0 min). Chlorophyll *a* and scytonemin were quantified by peak areas using the integrator of the software Agilent ChemStation Rev A.06.01 (Supplementary Figure S3).

To probe for the presence of expected or novel intermediary metabolites in the mutants we implemented a dual extraction/HPLC separation protocol, followed by fraction harvesting and mass spectroscopy of candidate peaks (see Supplementary Material for details).

### Bioinformatic and Phylogenetic Analyses

Orthologous sequences of cyanobacterial ScyA, ScyD, and ScyE proteins, and the respective genes coding for them, were retrieved at the NCBI (National Center for Biotechnology Information) database after Protein–Protein Basic Local Alignment Search Tool (BLASTp) analysis against the respective Scy proteins from *N. punctiforme*. All phylogenetic and molecular evolutionary analyses were conducted using MEGA version 6 (Tamura et al., 2013), after sequence alignment using ClustalW algorithms with the Gonnet protein weight matrix for amino acid alignments and the IUB DNA weight matrix for nucleotide alignments. Phylogenetic trees were constructed with the Minimum-Evolution, Neighbor-Joining, and Maximum-Likelihood methods, and evaluated with 500 bootstrap replicates. *N. punctiforme* NAD(P)H-quinone oxidoreductase subunit H (NdhH) was used as the outgroup sequence. The number of non-synonymous substitutions per non-synonymous site (*d*N) and the number of synonymous substitutions per synonymous site (*d*S) for *scyA*, *scyD*, and *scyE* in this dataset were calculated using different methods (see **Table 1**) (Jukes and Cantor, 1969; Li et al., 1985; Nei and Gojobori, 1986; Pamilo and Bianchi, 1993; Nei and Kumar, 2000).

**Table 1:** Evolutionary selection analyses of *scy* genes among the cyanobacterial radiation, as gauged by the ratio of the number of non-synonymous substitutions per non-synonymous site (*d*N) to the number of synonymous substitutions per synonymous site (*d*S), according to various algorithms.

Algorithm	*d*N/*d*S Ratio		
	*scyA*	*scyD*	*scyE*
Jukes and Cantor (1969)	0.22	0.97	0.61
Li et al. (1985)	0.24	0.90	0.57
Nei and Gojobori (1986)	0.40	0.99	0.72
Nei and Kumar (2000)	0.30	1.14	0.72
Pamilo and Bianchi (1993)	0.27	1.15	0.65

*In silico* prediction of signal peptides within ScyD, ScyE, and ScyF sequences was performed using the SignalP 4.1 Server^1^ (Petersen et al., 2011). Reanalysis of protein domains was carried out with the UniProt database (Universal Protein Resource), a comprehensive resource for protein sequence and annotation data (The UniProt Consortium, 2015).

## Results

### Characterization of *N. punctiforme scyD*, *scyE*, and *scyF* Knockout Mutant Strains

In order to assess the specific role of ScyD, ScyE, and ScyF in the scytonemin biosynthetic pathway, we constructed in-frame deletion mutants in which part of a gene is deleted but the rest of the operon is conserved in its proper reading frame (see **Figure 1B** and Supplementary Table S1 for details on construction). The DNA fragments used to generate the mutants were sequenced to guarantee that no other mutations were created during the construction. Chromosome segregation of the deletion mutants was confirmed by PCR (see Supplementary Figure S1 and Supplementary Table S2), using different combinations of oligonucleotides that flank the deleted region (so that smaller PCR products than those in the WT were expected, and observed), or that anneal to DNA regions that had been deleted (resulting in no PCR product). Examination under the optical microscope did not reveal any morphological differences, as defects in shape and size of the cells or differences in the length of the filaments, among these mutant strains grown under either white light or white light supplemented with UV-A radiation.

**FIGURE 2:**
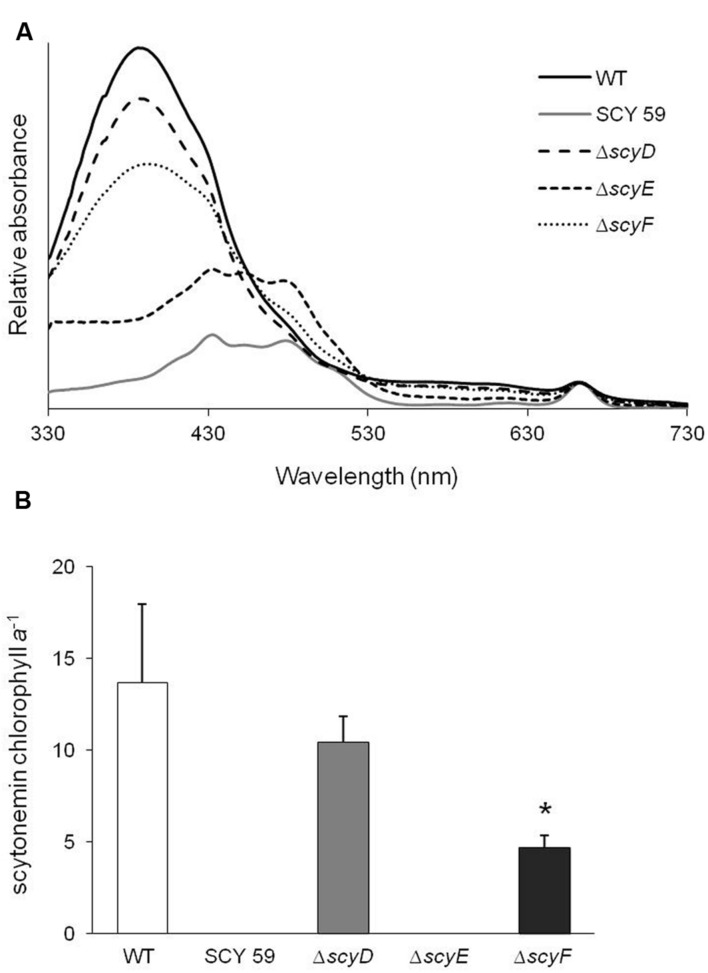
**(A)** Absorption spectra of lipid-soluble pigment extracts of UV-A induced cells of *N. punctiforme* wild-type (WT) and its derived mutants SCY 59, *ΔscyD*, *ΔscyE*, and *ΔscyF*. The characteristic peak at 384 nm indicates the presence of scytonemin. **(B)** Scytonemin to chlorophyll *a* ratios in extracts of WT and mutant strains after 6 days of UV-A induction, as quantified by HPLC (Supplementary Figure S3). The asterisk mark (*) indicates that the scytonemin/chlorophyll *a* ratio in *ΔscyF* is significantly different than the WT (*P* < 0.05). Error bars correspond to standard deviations from five experimental replicates.

To determine whether or not these strains were capable of producing scytonemin, cultures of WT and its derived deletion mutants *ΔscyD*, *ΔscyE*, *ΔscyF*, and a previously obtained scytoneminless mutant, SCY 59 (Soule et al., 2007) were subjected to a standard test for UV-A induction (Garcia-Pichel and Castenholz, 1991), and their lipid-soluble extracts were compared by spectroscopy. Only *ΔscyE* presented a scytoneminless phenotype like that known, and confirmed here, for SCY 59. Surprisingly, however, *ΔscyD* and *ΔscyF* extracts showed a strong absorption peak at 384 nm, indicative of scytonemin accumulation in the cells, together with the WT (**Figure 2A**). HPLC analyses confirmed the identity of scytonemin by its characteristic absorption maxima corresponding to the appropriate retention time of true standards. HPLC analyses were used to quantify chlorophyll *a* and scytonemin (Supplementary Figure S3 depicts a representative case). In addition, the inspection of UV-A induced *ΔscyD* and *ΔscyF* cells under the optical microscope did not reveal any differences in terms of subcellular localization of scytonemin in the mutants compared to the WT: in all three strains the pigment was clearly extracellular (Supplementary Figure S2).

Thus, among the three downstream genes in the “core biosynthetic area” of the operon, only *scyE* could in fact potentially code for a true biosynthetic gene in the pathway. If ScyE is responsible for picking up biosynthesis where ScyC left it, then the product of ScyC (compound 2 from **Figure 1A**), with molecular formula C_18_H_15_NO_2_ and found mass [M + H]^+^ 278.12 (Balskus and Walsh, 2009; Malla and Sommer, 2014), should be a substrate for ScyE, and a *scyE* knockout mutant should accumulate this substrate under conditions of UV induction. When we performed HPLC analyses in the extracts of UV-A induced *ΔscyE*, we could not detect the accumulation of such compound nor of its oxidized product. We only detected the accumulation of a novel peak (Supplementary Figure S4A) with absorption maxima at ca. 198, 231, 280, and 407 nm. However, when analyzing the collected fraction by mass spectrometry (MS), the compound was found to be [M + H]^+^ *m/z* 171.11 (Supplementary Figure S5A), a mass quite below that expected at this point in the biosynthetic pathway.

Since the deletion of *scyD* and *scyF* did not impair scytonemin production, a comparative quantification of the scytonemin yield was performed to see if there were any subtler effects between WT and each mutant. The scytonemin/chlorophyll *a* ratio, as measured by HPLC (Supplementary Figure S3) under identical conditions of induction, was significantly lower in *ΔscyF* than in WT (*P* < 0.05), while the ratio in *ΔscyD* did not differ significantly from WT (**Figure 2B**). These comparisons used five experimental replicates and statistical significance was determined using a Student’s *t*-test (two-tailed distribution, independent, and unequal variance) to compare WT with each mutant, separately.

In fact, HPLC analyses of *ΔscyF* extracts showed that, concomitantly with scytonemin production, a novel peak (Supplementary Figure S4B) with absorption maxima at ca. 210 and 370 nm accumulated. This fraction was collected and after MS analysis it was found to contain three distinct compounds with the following masses [M – H]^–^: 288.07, 377.08, and 417.20 (Supplementary Figure S5B).

### *In Silico* Analysis of *N. punctiforme* ScyD, ScyE, and ScyF

An *in silico* analysis of ScyD, ScyE, and ScyF amino acid sequences performed according to SignalP (Nielsen et al., 1997) was used by Soule et al. (Soule et al., 2009b) to deduce the existence of N-terminal signal peptides in these proteins, and to postulate their periplasmic fate. An updated analyses using SignalP 4.1 (Petersen et al., 2011) confirmed the prediction of a signal peptide cleavage site in ScyD and ScyE (with D-scores of 0.794 and 0.767, respectively, with nominal cutoff of 0.570 for significance), but not in ScyF (D-score of 0.412 with nominal cutoff of 0.510 for significance). Additionally, an evaluation at the UniProt database (2015) shows that both ScyE and ScyF contain a PEP-CTERM domain (IPR013424). This motif has been identified in a wide range of bacteria at their C-terminus and been suggested to be a protein export sorting signal (Haft et al., 2006), often associated with exopolysaccharide excretion. Thus bioinformatics still points to ScyF translocation across the membrane. Interestingly, when we examined the ScyD C-terminal region more in detail, we were able to identify a typical Proline-Glutamic acid-Proline (PEP) motif but some of the other domain characteristics seem to be missing. Our most recent analysis of the putative gene products revealed that ScyF has additional NHL repeats (IPR001258), of which a domain subgroup (IPR013017) could be involved in protein–protein interactions (Edwards et al., 2003).

### Phylogenetic Analysis of ScyD and ScyE

**FIGURE 3:**
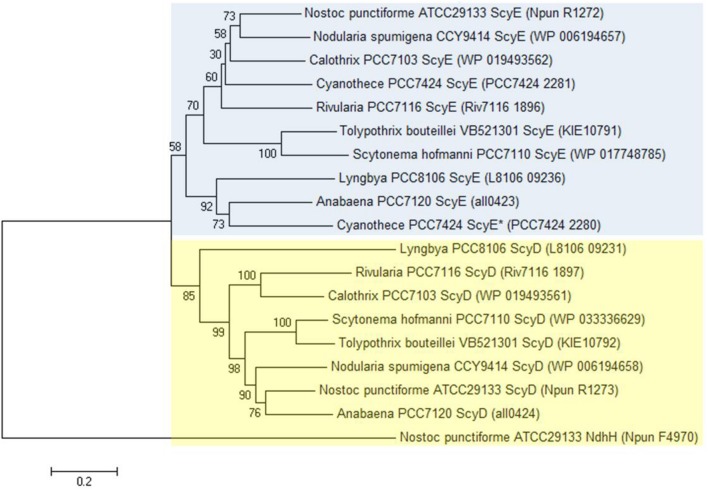
**Minimum Evolution Phylogenetic tree reconstructed on the basis of ScyD and ScyE amino acid sequences.** Phylogenetic trees were created with bootstrap values from 500 replicates. *N. punctiforme* NAD(P)H-quinone oxidoreductase subunit H (NdhH) was used as the outgroup sequence. This topology is supported by both Neighbor-Joining and Maximum-Likelihood methods (see Supplementary Figure S6). Blue box highlights the ScyE cluster, while the yellow box the ScyD. The GenBank accession number for each protein is given between brackets.

Even though we found ScyD and ScyE to have a dissimilar involvement in scytonemin production, the two proteins are quite similar in sequence. In fact, *scyD* seems to have originated as a duplication of *scyE* (Soule et al., 2007). Therefore, their phylogeny and molecular evolution were also investigated in more detail here. We chose cyanobacterial strains whose genomes are fully sequenced and where all six *scyABCDEF* genes are present. Nine strains were selected: *Anabaena* sp. 7120 (also known as *Nostoc* sp. PCC 7120), *Calothrix* sp. PCC 7103, *Cyanothece* sp. PCC 7424, *Lyngbya* sp. PCC 8106 (also known as *Lyngbya aestuarii* CCY 9616), *Nodularia spumigena* CCY 9414, *N. punctiforme* ATCC 29133 (PCC 73102), *Rivularia* sp. PCC 7116, *Scytonema hofmanni* PCC 7110 (ATCC 29171), and *Tolypothrix bouteillei* VB521301. Nonetheless, we realized that *Cyanothece* sp. PCC 7424 has two copies of ScyE instead of one of ScyE and one of ScyD; one of them shares 61% amino acid sequence identity with *N. punctiforme* ScyE (*Cyanothece* PCC 7424 ScyE), and the other one 44% (*Cyanothece* PCC 7424 ScyE*). Thus, all subsequent analyses were carried out by using eight homologs of ScyD and ten of ScyE. Amino acid phylogenetic trees reconstructed using several algorithms were consistent in presenting ScyD and ScyE as part of distinct clusters (**Figure 3** and Supplementary Figure S6).

We investigated nucleotide substitutions in the core genes of the scytonemin operon system to assess the degree of selection operating on each (Sorrels et al., 2009; Tai et al., 2011; Dvornyk and Jahan, 2012). We used the ratio of the number of non-synonymous substitutions per non-synonymous site (*d*N) and the number of synonymous substitutions per synonymous site (*d*S). If *d*N/*d*S < 1, the gene in consideration is under purifying selection, where non-synonymous amino acid altering mutations are selected against, likely as a means to maintain function. A *d*N/*d*S value around one will indicate neutral selection and a gene that is not under selective pressure. Values larger than one indicate positive (or Darwinian) selection. Purifying selection acts on the large majority of protein coding genes. In our analyses, *scyA*, essential to scytonemin biosynthesis and previously found to be under a tight selection that does not accommodate mutational changes (Sorrels et al., 2009), was used as a control. *d*N/*d*S ratios were calculated according to different algorithms (Jukes and Cantor, 1969; Li et al., 1985; Nei and Gojobori, 1986; Pamilo and Bianchi, 1993; Nei and Kumar, 2000) and found to be always well below one for *scyA* (ranging from 0.22 to 0.40) and *scyE* (0.57 to 0.72), but hovering around unity for *scyD* (0.90 to 1.14) (**Table 1**), indicating that the latter is not under significant selective pressure.

## Discussion

Since the discovery of the operon responsible for the scytonemin production in *N. punctiforme* by Soule et al. (2007), advances in understanding its molecular and biochemical properties were achieved through comparative genomics, *in vitro* studies and heterologous expression of its genes in *E. coli* (Balskus and Walsh, 2008; Balskus and Walsh, 2009; Sorrels et al., 2009; Soule et al., 2009b; Malla and Sommer, 2014). Such reports helped to elucidate the role of some proteins, like ScyA, ScyB, and ScyC, or the non-housekeeping homologs of aromatic amino acids biosynthesis genes co-transcribed downstream to supplement the initial building blocks. Furthermore, these studies were essential for the formulation of some predictions, such as the putative regulatory role of the upstream response regulator and histidine kinase (**Figure 1B**) (Sorrels et al., 2009; Soule et al., 2009b), and the assumption that ScyD, ScyE, and ScyF are responsible for late steps in the biosynthetic pathway and, perhaps the most intriguing, that such putative enzymes, and hence the late pathway, reside in the periplasm (Soule et al., 2009b; Gao and Garcia-Pichel, 2011). The results obtained in the present study are sufficient to reject the hypothesis on the role of *scyDEF*.

From all in-frame deletion mutants of each gene we constructed, only *ΔscyE* resulted in a scytoneminless phenotype. Clearly, neither ScyD nor ScyF are required for full scytonemin synthesis (**Figure 2**). It may seem paradoxical that the transposon mutagenesis effort (Soule et al., 2007) that yielded the first scytoneminless mutant, SCY 59, and pointed to this locus altogether, was traced to *scyD*. However, one has to bear in mind that the insertion of the antibiotic resistance cassette in mutant SCY 59 disrupted not only the expression of *scyD*, but also the entire set of genes downstream of it. In the light of our results, we interpret that the phenotype of SCY 59 is possibly due to a polar effect on the downstream proteins.

Nevertheless, the finding that the protein encoded by *scyD* does not play a role in the scytonemin biosynthesis is still puzzling due to its high conservation among cyanobacteria, where it occupies a central position in the operon (Soule et al., 2009b). In addition, two independent studies have previously shown that *scyD* transcript levels are also up-regulated after induction by UV-A radiation exposure, along with the transcription of the other genes within the 18 cluster (Sorrels et al., 2009; Soule et al., 2009a). The contention that *scyD* might contain regulatory RNAs could be put forth, but we did not find any such predicted structures to support it. Other examples of transcription of non-functional genes (pseudogenes) that are part of functional operons in bacteria are found in the literature, possibly due to the conservation of read-through transcription as a result of their location within the operon (e.g., Forest et al., 2007; Williams et al., 2009; Feng et al., 2011). It was also clear from the phylogenetic reconstructions that ScyD and ScyE belong to distinct clusters (**Figure 3**), in agreement with the origin of one in an ancestral duplication event of the other (Soule et al., 2007) that lead to dissimilar functions. If there is indeed a differentiated function by ScyD, that function is not a requirement for scytonemin synthesis. Moreover, protein evolutionary analyses showed that, unlike the genes required for scytonemin production, *scyA* and *scyE*, *scyD* has not been subjected to purifying selection (**Table 1**), indicating that its putative translated product is not functional. In this context, we may consider *scyD* to be a pseudogene (Li et al., 1981; Vanin, 1985; Zheng et al., 2005), even though we must admit that such pseudogene would have been kept in place within the long evolutionary history spanning a larger portion of the cyanobacterial radiation, likely billions of years, which seems in itself paradoxical. Notwithstanding, there are some exceptions to the conservation of *scyD* within the scytonemin operon, for example, in *Chroococcidiopsis thermalis* PCC 7203 *scyD* (WP_015153365) is not located in the same genomic region as *scyABCEF* (WP_015155130 to WP_015155134), and we found *Cyanothece* PCC 7424 to contain two copies of *scyE* and none of *scyD*. Considering all this information, we suggest that the duplication leading to *scyD* was redundant but it has been kept within the *scy* operon of most cyanobacteria due to its central physical location, and to the fact that it is flanked by two genes essential for scytonemin biosynthesis (*scyC* and *scyE*).

As expected, *ΔscyE* presented a scytoneminless phenotype (**Figure 2**) but none of its potential substrates seem to accumulate in significant quantities. In particular, no accumulation of the ketone catalyzed by ScyC (compound 2 in **Figure 1A**, [M + H]^+^ *m/z* 278.12), nor its potential oxidized product, the monomer moiety of scytonemin (compound 3 in **Figure 1A**, [M + H]^+^ *m/z* 276.10) (Malla and Sommer, 2014), were detected. However, we did distinguish the accumulation of small quantities of a *ΔscyE* exclusive compound with [M + H]^+^ *m/z* 171.11, a mass much smaller than expected. We cannot exclude the possibility that compound 2 is toxic to *N. punctiforme*’s cells, thus being quickly degraded into the compound found [M + H]^+^ 171.11, though this would be quite unlikely because an oxidized version of compound 3 is found in *Nostoc* spp. strains (Kobayashi et al., 1994; Ploutno and Carmeli, 2001). Consistent with our findings, Malla and Sommer (2014) showed that, upon supplementation of tryptophan and tyrosine, *E. coli* produces the same monomer moiety of scytonemin when expressing either *scyABC* or *scyABCDEF*. Altogether, these data confirm that the *scyE* position in the operon does not mirror the biosynthetic sequence of reactions (ScyE is not involved in the oxidation of the ScyC product), and indicate that ScyE is not involved in the last steps of scytonemin biosynthesis in a direct manner. Unfortunately, *in silico* analysis of ScyE conserved domains does not offer clear suggestions as to its role, as it is part of the beta-propeller clan, a protein family that is functionally quite heterogeneous. ScyE can be predicted to be periplasmic on the basis of the presence of putative signal peptides (Soule et al., 2007, 2009b; this study). It has been shown that cyanobacteria can export proteins across the cytoplasmic membrane through the Sec and the Tat pathways using such signal peptides (Liberton and Pakrasi, 2008; Barnet et al., 2011). SignalP 4.1 (Petersen et al., 2011), supports the prediction of a ScyE translocation through the Sec system. The exact role of ScyE, while crucial for scytonemin biosynthesis, remains to be established.

*ΔscyF* showed a reduction of the sunscreen yield compared with the WT and it accumulated an uncharacterized product only in cultures exposed to UV-A radiation (**Figure 2B** and Supplementary Figure S4B). This suggests that ScyF is not essential but might be an adjuvant for the scytonemin synthesis. A UniProt database (2015) analysis shows that the ScyF amino acid sequence contains a NHL domain subgroup (IPR013017) that could be involved in protein–protein interactions (Edwards et al., 2003). We can speculate that ScyF is a chaperone that helps the folding of an enzyme involved in the scytonemin synthetic pathway. On the other hand, it is also possible that ScyF is the catalyst of an otherwise spontaneous reaction, or that it suppresses the formation of an off-pathway reaction, in a manner similar to ScyC, which steers the pathway away from a non-enzymatic decarboxylation of the ScyA product, that would result in the production of an α-hydroxyketone regioisomer (Balskus and Walsh, 2009).

## Conclusion

Our findings suggest that the proteins encoded by *scyDEF*, the region downstream of the known scytonemin biosynthetic core *scyABC*, are not, as predicted, responsible for the late reactions in the biosynthetic pathway. It is clear that ScyA, ScyB, and ScyC are not sufficient for the pigment biosynthesis. Therefore, one must look elsewhere in the genome for the missing late enzymes. A comparative genomics study discovered that in most cyanobacteria the scytonemin operon contains a group of five genes of unknown function and high phylogenetic conservation, placed typically immediately downstream of the *scyDEF* region (Soule et al., 2009b). In *N. punctiforme* and closely related strains these genes are also present, but located outside of the main operon as a satellite five-gene cluster (Npun_F5232 to Npun_F5236). We consider these to be good candidates, and will be investigating their possible involvement in the near future.

## Author Contributions

FG-P conceived this study; DF and FG-P designed the experiments; DF performed the experimental work; manuscript was written by DF with help by FG-P. All authors have read and approved the final manuscript.

## Conflict of Interest Statement

The authors declare that the research was conducted in the absence of any commercial or financial relationships that could be construed as a potential conflict of interest.

## Footnotes

1 http://www.cbs.dtu.dk/services/SignalP/
