# Supplementary material for: Mutational Studies of Putative Biosynthetic Genes for the Cyanobacterial Sunscreen Scytonemin in Nostoc punctiforme ATCC 29133
Source: Front Microbiol. 2016 May 18;7:735. doi: 10.3389/fmicb.2016.00735 (PMC4870267; doi:10.3389/fmicb.2016.00735)
Supplement: Supplementary file 1 [file Data_Sheet_1.DOCX]

Supplementary Material

Mutational studies of putative biosynthetic genes for the cyanobacterial sunscreen scytonemin in *Nostoc punctiforme* ATCC 29133

**Daniela Ferreira, Ferran Garcia-Pichel***

***Correspondence:** Ferran Garcia-Pichel: ferran@asu.edu

**Material and Methods**

**Attempts to identify scytonemin-related precursors**

Lipid-soluble pigment extracts from *N. punctiforme* wild-type (WT) and its derived deletion mutants, SCY 59, *∆scyD*, *∆scyE*, and *∆scyF*, UV-A induced cells were obtained as described in the main Material and Methods section. The extracts were analyzed by three different high pressure liquid chromatography (HPLC) methods, using an Agilent 1100 HPLC system with an online photodiode array detector. The column and method described by Soule *et al.* (2007) were first used with 100 µl of concentrated acetone extracts. Absorption spectra of the pigments were monitored at 384 nm but also recorded continuously between 330-700 nm. Chlorophyll *a*, carotenoids and scytonemin were identified by their characteristic absorption maxima corresponding to the appropriate retention time, but no new peaks in the deletion mutants extracts could be identified by this method. Consequently, the HPLC procedures described by Karsten and Garcia-Pichel (1996), as well Malla and Sommer (2014), were followed. In the latter, an extra step was added after the run (30-33 min 25-100% acetonitrile, 33-53 min 100%, 53-58 min 100-25% acetonitrile, and 58-60 min 25% acetonitrile), in order to clean the column from cyanobacterial residues of chlorophyll *a* and carotenoids. Absorption spectra of pigments were recorded continuously between 190-700 nm. Chlorophyll *a*, carotenoids and scytonemin were again recognized by their characteristic retention time and absorption maxima. Even though both HPLC methods were used in all the WT and mutant strains, new uncharacterized peaks were identified for the extracts of *∆scyE* at 18.58 min when using the protocol described by Malla and Sommer, 2014, and for *∆scyF* at 1.06 min when using the protocol by Karsten and Garcia-Pichel, 1996 (Fig. S4). Subsequently, these peaks were collected five times with a Gilson FC 205 Fraction Collector using independent biological replicates and concentrated.The exact mass of the compound(s) was analyzed by mass spectrometry using a Bruker Daltonics micrOTOF-Q in the positive and negative ion modes. Fractions collected from the wild-type at the same respective time and solvents utilized to run the HPLC were used as negative controls for the MS. Thus, only peaks found exclusively in the fractions collected for the mutants were considered (Fig. S5).

Table S1. *N. punctiforme* strains used in this work.

| *N. punctiforme* strains | Relevant characteristics |
| --- | --- |
| WT | UCD 153, a ATCC 29133 wild-type derivate, scytonemin producer (Campbell *et* *al.*, 2007) |
| SCY 59 | *scyD*::Tn5-1063a, scytoneminless mutant, Nm^r^ (Soule *et al.*, 2007) |
| *∆scyD* | *scyD* knockout  Primers used to create a 1044 bp deletion: scyDL-XhoI: ATCGCGCTCGAGACACACGAGCATCCCAATCTTCC, scyDI1: AGCTGACGCAGCTTTCATTCCAG, scyDI2: CTGGAATGAAAGCTGCGTCAGCTCGCCCTGGATTTGGACAAGTTCTC, scyDR: CTTGGAATTGCCCAGGTGCAGTG |
| *∆scyE* | *scyE* knockout  Primers used to create a 1014 bp deletion: scyEL: GGAACTGCATCGGGTTATCAC, scyEI1: ACCATCGGCAACTACTGTTAGG, scyEI2: CCTAACAGTAGTTGCCGATGGTTATATCGCAAACCGAGCCAGAC, scyER: CGGCAATGACGCTAATGGTATG |
| *∆scyF* | *scyF* knockout  Primers used to create a 882 bp deletion: scyFL: ATGCTGACGGGAGAAATCTG, scyFI1: GCTATCCACCGTTATGCCTTG, scyFI2: CAAGGCATAACGGTGGATAGCGTAGAGGGCAGAAAGCAAGTACC, scyFR: CCATCATCCGATCGTGTTCTTCAG |

Underlined letters indicate introduced restriction endonuclease cleavage sites.

Table S2. Oligonucleotides used in this study to confirm homozygosity of the in-frame deletion mutants.

| Oligonucleotide | Sequence | PCR reaction*^a^* |
| --- | --- | --- |
| scyDF | ATCGCGTCTAGAAACTCCTCCCTCAATTCCTTGC | D1 and D3 |
| scyDrev | GTTGAGATTTATGGGAGGTGTGGG | D2 and D3 |
| scyDR0 | TGCGTTCGGTCTTACCATTCTC | D1 |
| scyFL | ATGCTGACGGGAGAAATCTG | D2 |
| scyEF | CCGCCGTCTAGAGCTGGGATTCGTTGTCTTTAAACC | E1 and E3 |
| scyErev | GACAGTCTCTGCTTTCACTAATTC | E2 and E3 |
| scyER0 | ATATACCCTGCGCTAGGAGATG | E1 |
| scyEF0 | GACCCTGACGGCAATTTGTACG | E2 |
| scyFF | CCGCCGTCTAGATAGTCTAGGAACGAGTATACCTC | F1 and F3 |
| scyFrev | GCATTGCTTTTGCAGTTCTTTCTC | F2 and F3 |
| scyFR0 | CCAATATACTGGCCGCTTGGAC | F1 |
| scyFF0 | GGTACCGCCTCCAGCATTAAC | F2 |

*^a^* PCR reactions used as illustrated in Fig. S1


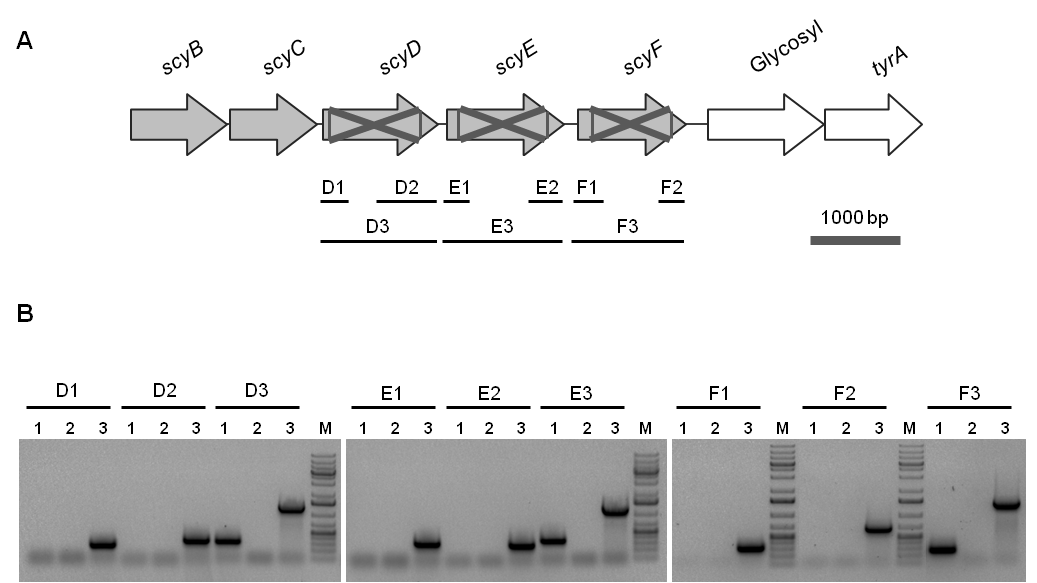


Figure S1. Confirmation of *scy* individual mutant clones in *N. punctiforme*. (A) Partial scytonemin biosynthesis genomic region drawn to scale depicting the in-frame deletion mutants constructed in this study. The arrows represent ORFs and indicate the transcriptional direction. Grey ORFs represent scytonemin core genes and white ORFs the genes associated with aromatic amino acid biosynthesis. The grey cross and vertical grey lines inside the ORFs show the exact location of the gene deletion. The black horizontal lines bellow the physical map indicate the PCRs performed in wild-type and mutants with primers covering or flanking the deleted regions (see supplementary Table S2). (B) Identity and homozygosity of mutants tested by PCR using three combinations of oligonucleotides for each construction (see Table S2 for oligonucleotide details). Mutant PCR products were not detected or they were smaller than wild-type products. Lane 1: deletion mutant, lane 2: negative control (water), lane 3: wild-type control, lane M: marker.


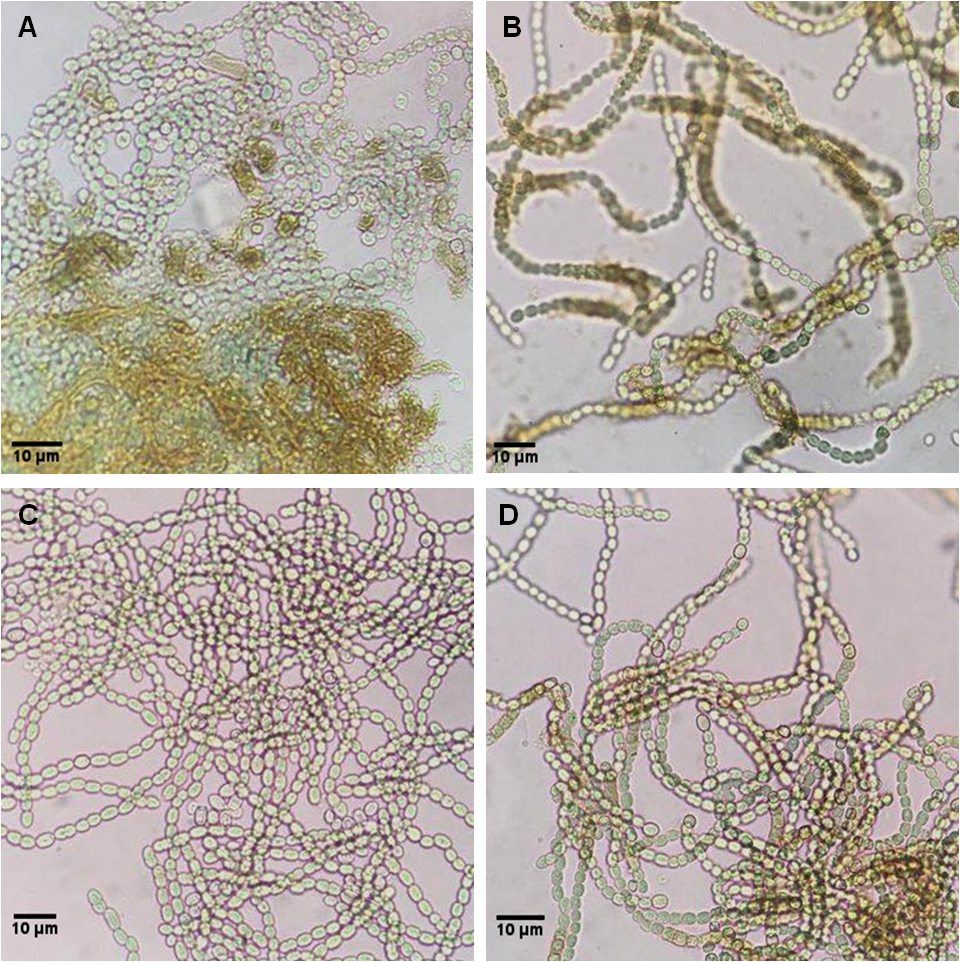


Figure S2. Photomicrographs of *N. punctiforme* (A) wild-type and its derived mutants (B) *∆scyD*, (C) *∆scyE*, and (D) *∆scyF* cells after UV-A induction.


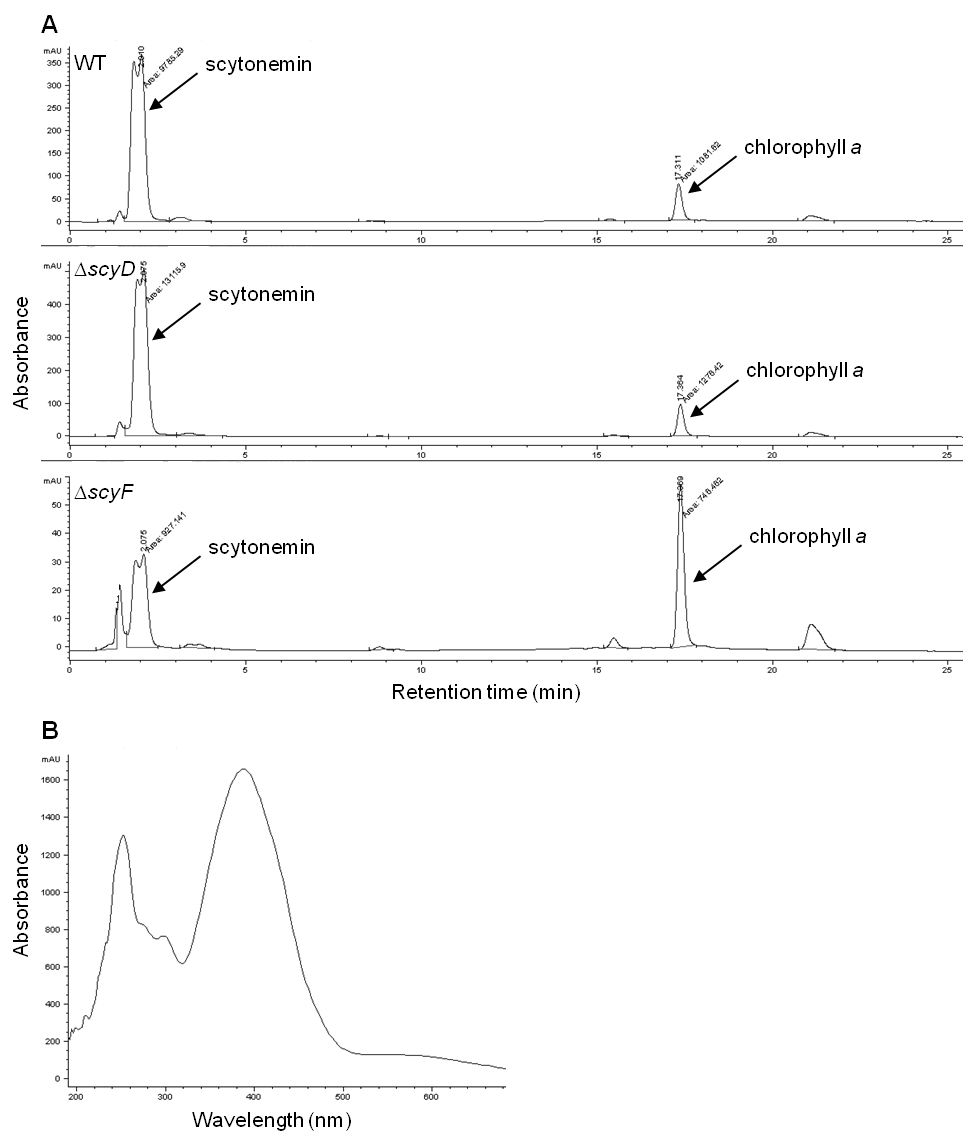


Figure S3. (A) Representative image for HPLC chromatograms of *N. punctiforme* wild-type (WT) and its derived mutants *∆scyD* and *∆scyF* lipid-soluble pigment extracts from UV-A induced cells, as described by Karsten and Garcia-Pichel (1996) and recorded at 384 nm. The arrows indicate scytonemin and chlorophyll *a* accumulation, subsequently quantified by peak areas. The assays were repeated with five biological replicates and used to generate the data depicted on Fig. 2B. (B) Example of an absorption spectrum for scytonemin-containing WT, as recorded between 190-700 nm by HPLC system with an online photodiode array.


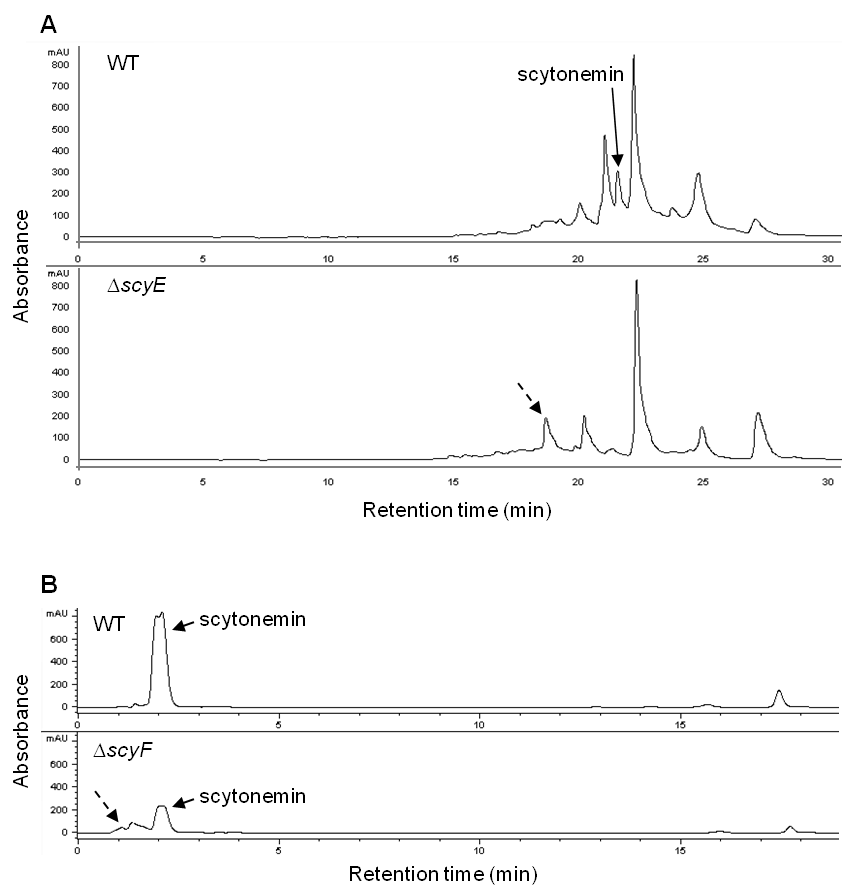


Figure S4. HPLC chromatograms of *N. punctiforme* wild-type (WT) and its derived mutants *∆scyE* and *∆scyF* lipid-soluble pigment extracts from UV-A induced cells. The images shown here were chosen to depict the identification of uncharacterized peaks for *∆scyE* and *∆scyF* extracts. HPLC method described by (A) Malla and Sommer (2014) as recorded at 407 nm, and (B) Karsten and Garcia-Pichel (1996) as recorded at 384 nm. Solid arrows indicate scytonemin accumulation, while dashed arrows indicate the presence of uncharacterized peaks, not present in the other extracts, which were collected and analyzed by MS (Fig. S5).


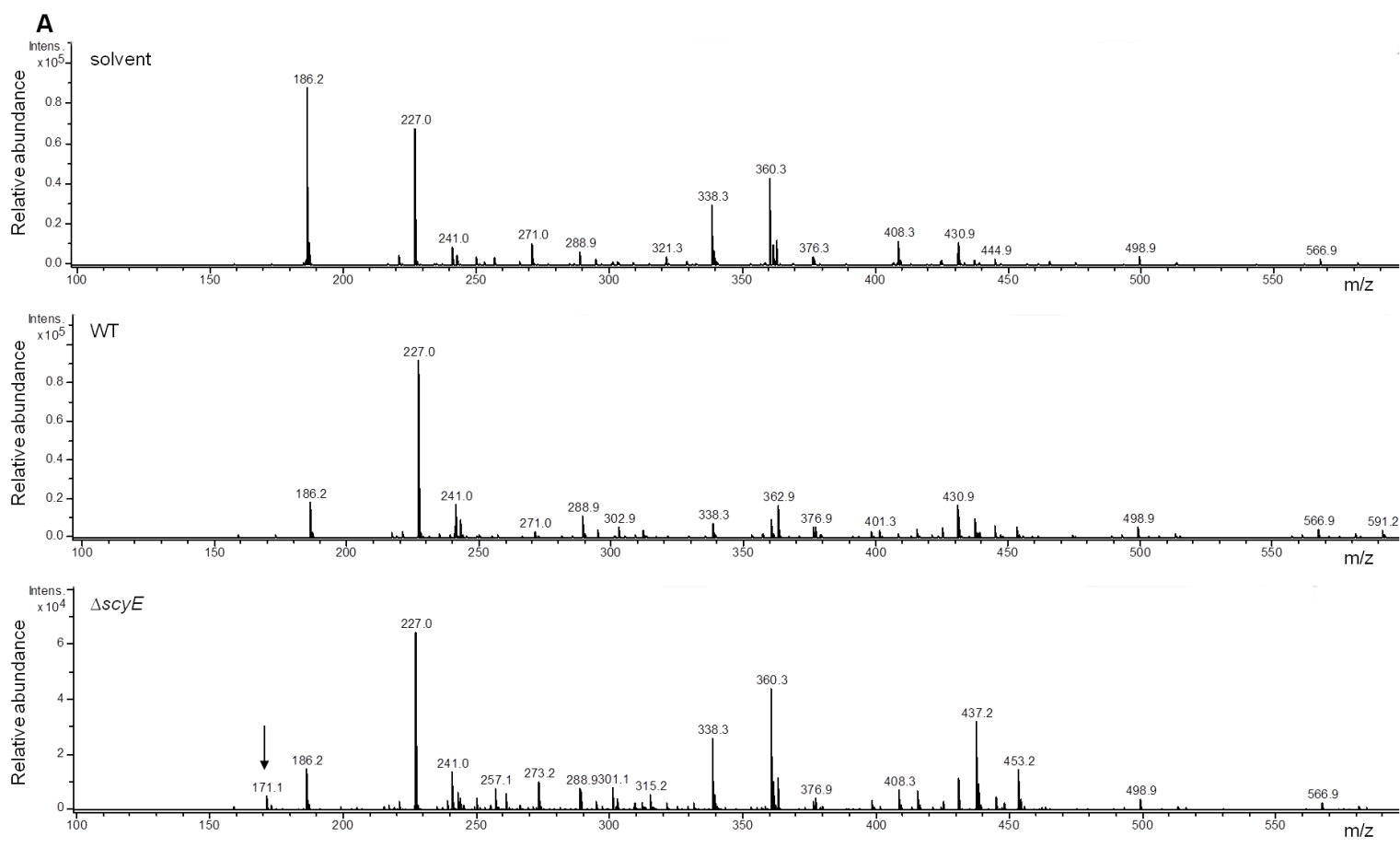


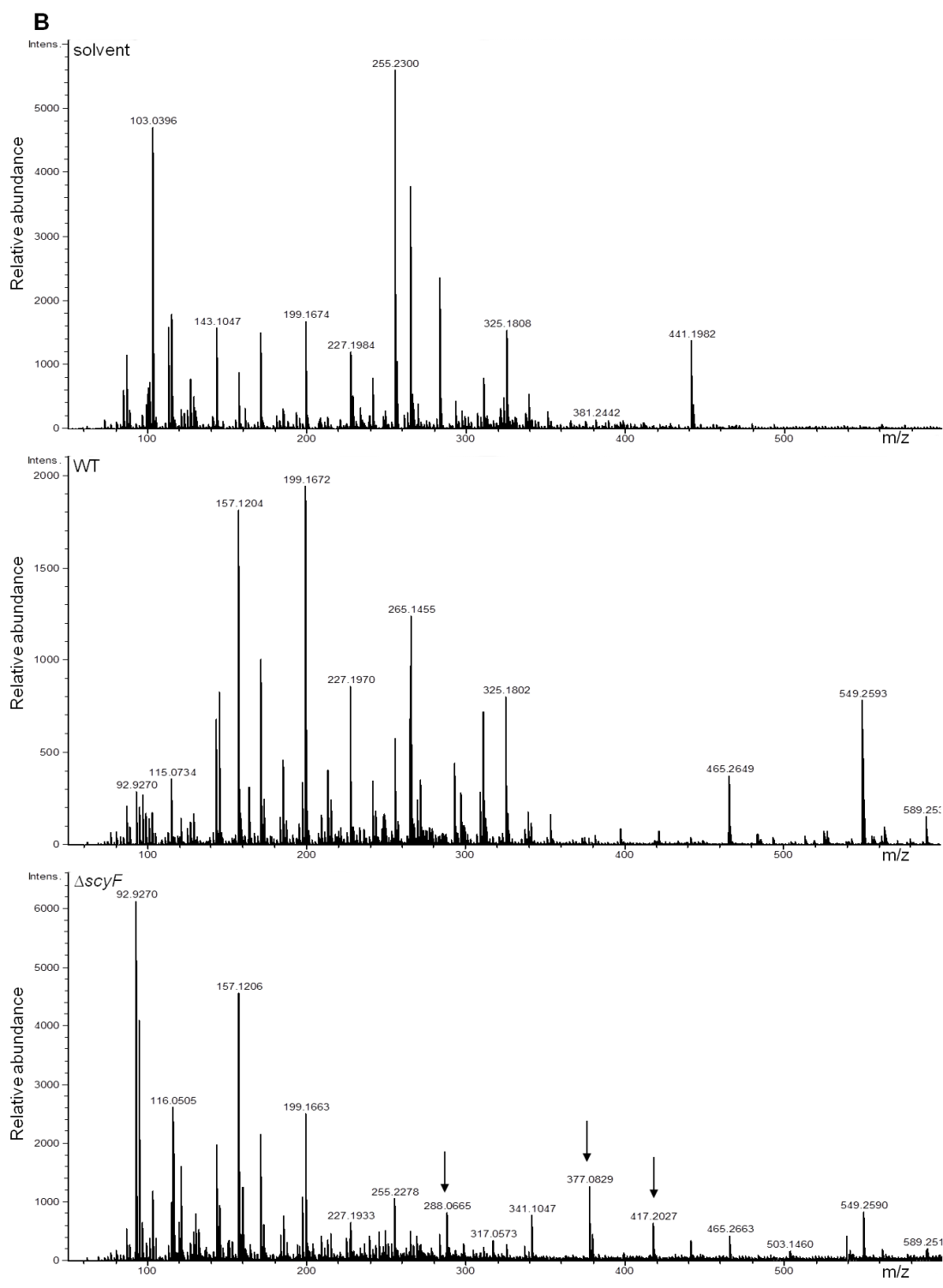


Figure S5. MS analysis of metabolites from *N. punctiforme* mutants, *∆scyE* and *∆scyF*, after collection of HPLC fractions (see Fig. S4). Solvents utilized to run the HPLC and fractions collected from wild-type (WT) at the same respective time were used as negative controls. Solid arrows indicate the presence of peaks that were not present in the negative controls (A) exact mass found for *∆scyE* compound in the positive mode: [M + H]^+^ *m/z* 171.11 (B) exact masses found for *∆scyF* compounds in the negative mode: [M - H]^-^ *m/z* 288.07, 377.08 and 417.20.


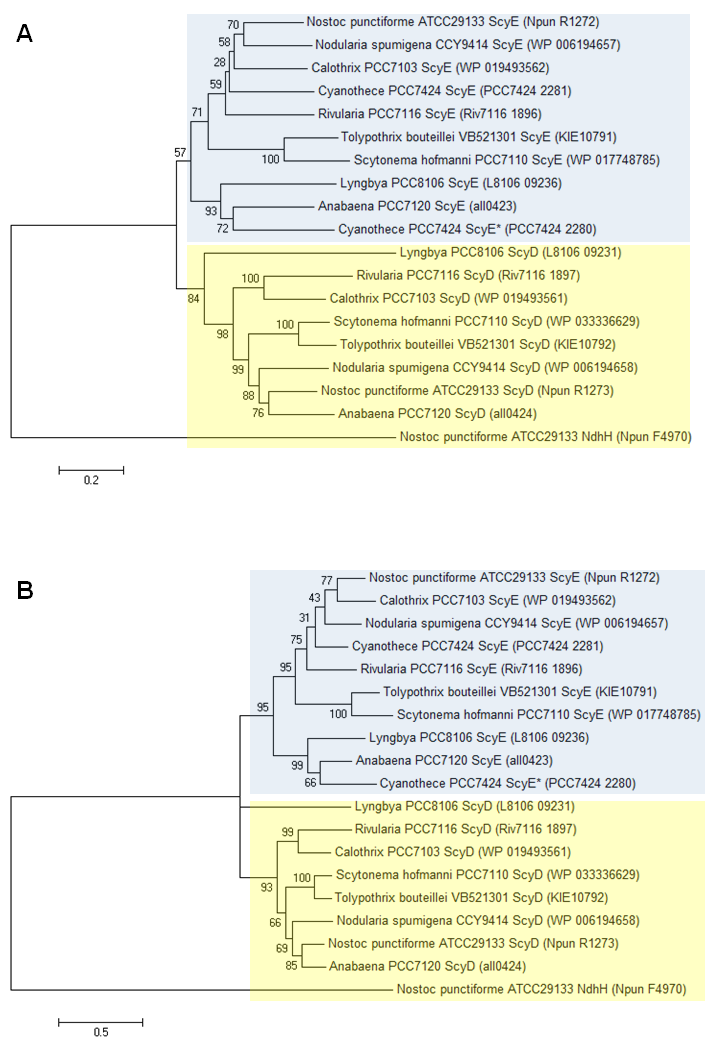


Figure S6. Phylogenetic trees reconstructed on the basis of ScyD and ScyE amino acid sequences. Phylogenetic trees were created with the (A) Neighbor-Joining or (B) Maximum Likelihood methods with bootstrap values from 500 replicates. *N. punctiforme* NAD(P)H-quinone oxidoreductase subunit H (NdhH) was used as the outgroup sequence. Blue box represents the ScyE cluster, while the yellow box the ScyD. The GenBank accession number for each protein is between brackets.
